# Draft Genome Sequence of the Type Species of the Genus *Citrobacter, Citrobacter freundii* MTCC 1658

**DOI:** 10.1128/genomeA.00120-12

**Published:** 2013-01-31

**Authors:** Shailesh Kumar, Chandandeep Kaur, Kazuyuki Kimura, Masahiro Takeo, Gajendra Pal Singh Raghava, Shanmugam Mayilraj

**Affiliations:** a Microbial Type Culture Collection and Gene Bank (MTCC), Chandigarh, India; b Bioinformatics Centre, CSIR-Institute of Microbial Technology, Chandigarh, India; c Department of Materials Science and Chemistry, Graduate School of Engineering, University of Hyogo, Himeji, Hyogo, Japan

## Abstract

We report the 5.0-Mb genome sequence of the type species of the genus *Citrobacter*, *Citrobacter freundii* strain MTCC 1658, isolated from canal water. This draft genome sequence of *C. freundii* strain MTCC 1658^T^ consists of 5,001,265 bp with a G+C content of 51.61%, 4,691 protein-coding genes, 70 tRNAs, and 10 rRNAs.

## GENOME ANNOUNCEMENT

The genus *Citrobacter* was first proposed as *Citrobacter freundii* (Braak 1928) by Werkman and Gillen (1). At present, the genus *Citrobacter* consists of ten recognized species, including *C. freundii* MTCC 1658. The type species *C. freundii* MTCC 1658 is a Gram-negative aerobic short-rod bacterium isolated from canal water in the United States. The genome of strain MTCC 1658^T^ was sequenced using a standard run of the Roche 454 sequencing technology. A total of 891,216 reads with an average length of 402.52 nucleotides (~71.7-fold coverage) were used for genome assembly by the Newbler assembler version 2.5.3 (Roche) using the default parameters. A total of 171 contigs of >500 bp in length were constructed, with an *N*_50_ of 196.05 kb; the largest contig assembled measured 445.73 kb. The final draft genome sequence consists of 5,001,265 bp, and the Rapid Annotations using Subsystems Technology (RAST) server (2) and tRNAscan-SE-1.23 software (3) were used for genome annotation.

Whole-genome annotation with the RAST server shows that strain MTCC 1658^T^ contains genes for urea decomposition (i.e., genes for urease accessory proteins UreD, UreE, UreF, and UreG), chitin and *N*-acetylglucosamine utilization, dehydrogenase complexes, dihydroxyacetone kinases, the Entner-Doudoroff pathway, glycolysis and gluconeogenesis, pentose phosphate pathways, the tricarboxylic acid (TCA) cycle, lactose and galactose uptake and utilization, sialic acid metabolism, the riboflavin synthesis cluster, polysaccharide export of lipoprotein Wza, the pyruvate metabolism I system (anaplerotic reactions and phosphoenolpyruvate [PEP]), and another pyruvate metabolism system (acetyl coenzyme A [acetyl-CoA] and acetogenesis from pyruvate). Also, we found the genes for isocitrate lyase (EC 4.1.3.1), malate synthase (EC 2.3.3.9), biofilm PGA (polyglycolic acid) synthesis *N*-glycosyltransferase PgaC (EC 2.4.-.-), GlmU, GlmM, and GlmS. We have also found the genes for arginine deaminase (EC 3.5.3.6), proline iminopeptidase (EC 3.4.11.5), *N*-acetyl-d-glucosamine ABC transport system permease protein 1, *N*-acetyl-d-glucosamine ABC transport system permease protein 2, and galactosamine-6-phosphate isomerase (EC 5.3.1.-).

### Nucleotide sequence accession numbers. 

This Whole Genome Shotgun project has been deposited at DDBJ/EMBL/GenBank under the accession no. ANAV00000000. The version described in this article is the first version, ANAV01000000. Genome assembly and annotation data can be downloaded from our genomics web portal at http://crdd.osdd.net/raghava/genomesrs/.
